# Androgen suppresses protein kinase D1 expression through fibroblast growth factor receptor substrate 2 in prostate cancer cells

**DOI:** 10.18632/oncotarget.14536

**Published:** 2017-01-06

**Authors:** Liyong Zhang, Zhenlong Zhao, Shuping Xu, Manuj Tandon, Courtney R. LaValle, Fan Deng, Q. Jane Wang

**Affiliations:** ^1^ Department of Pharmacology and Chemical Biology, University of Pittsburgh School of Medicine, Pittsburgh, PA, USA; ^2^ Department of Anesthesiology, Nanfang Hospital, Southern Medical University, Guangzhou, Guangdong, China; ^3^ Department of Cell Biology, School of Basic Medical Sciences, Southern Medical University, Guangzhou, Guangdong, China

**Keywords:** androgen, androgen-repressed genes, androgen receptor, protein kinase D1, prostate cancer

## Abstract

In prostate cancer, androgen/androgen receptor (AR) and their downstream targets play key roles in all stages of disease progression. The protein kinase D (PKD) family, particularly PKD1, has been implicated in prostate cancer biology. Here, we examined the cross-regulation of PKD1 by androgen signaling in prostate cancer cells. Our data showed that the transcription of PKD1 was repressed by androgen in androgen-sensitive prostate cancer cells. Steroid depletion caused up regulation of PKD1 transcript and protein, an effect that was reversed by the AR agonist R1881 in a time- and concentration-dependent manner, thus identifying PKD1 as a novel androgen-repressed gene. Kinetic analysis indicated that the repression of PKD1 by androgen required the induction of a repressor protein. Furthermore, inhibition or knockdown of AR reversed AR agonist-induced PKD1 repression, indicating that AR was required for the suppression of PKD1 expression by androgen. Downstream of AR, we identified fibroblast growth factor receptor substrate 2 (FRS2) and its downstream MEK/ERK pathway as mediators of androgen-induced PKD1 repression. In summary, PKD1 was identified as a novel androgen-suppressed gene and could be downregulated by androgen through a novel AR/FRS2/MEK/ERK pathway. The upregulation of prosurvival PKD1 by anti-androgens may contribute to therapeutic resistance in prostate cancer treatment.

## INTRODUCTION

Prostate cancer is the most common noncutaneous malignancy and the second leading cause of cancer-related deaths among men in the United States. The initiation and progression of prostate cancer is uniquely dependent on androgen receptor (AR)-induced signaling. Although androgen deprivation therapy provides an initial favorable response in advanced prostate cancer, the more aggressive castration-resistant prostate cancer (CRPC) develops invariably in almost all patients, eventually leading to death. It has become increasingly clear that continuous activation of the AR in CRPC remains the main driving force of tumor progression and metastasis. Thus, understanding the critical events associated with the AR signaling is essential for developing novel and effective therapies to treat CRPC.

The protein kinase D (PKD) family of serine/threonine kinases belongs to the Ca^2+^/calmodulin-dependent protein kinase (CAMK) superfamily [1, 2]. To date, three isoforms of PKD have been identified, PKD1 (formerly PKCμ) [3, 4], PKD2 [5], and PKD3 (formerly PKCν) [6]. In intact cells, PKD activation involves phosphorylation of two conserved serine residues in the activation loop by DAG-responsive PKCs [7–9], and PKD activity can be maintained independently of PKC through autophosphorylation [10, 11]. Emerging evidence supports that PKD has an important role in carcinogenesis and tumor progression [12, 13]. A recent report suggested that a hotspot activating mutation in *PRKD1*, the gene encoding PKD1, may drive polymorphous low-grade adenocarcinoma (PLGA), the second most frequent type of malignant tumor of the minor salivary glands [14]. PKD regulates a variety of tumor-associated biological processes, including tumor cell proliferation, growth, survival, migration, invasion, secretion, and angiogenesis [12, 15–20]. Aberrant PKD activity and expression have been demonstrated in tumor cell lines and tumor tissues from the pancreas [18], skin [19, 21], breast [22], and prostate [20, 23]. In particular, PKD has been shown to play an important role in the pathogenesis of prostate cancer [20, 24–26], and targeted PKD inhibition potently blocks prostate cancer cell proliferation and survival [26, 27].

Fibroblast growth factor (FGF) signaling is a highly complex signaling network that comprises 18 ligands, which bind to and activate four highly conserved transmembrane tyrosine kinase receptors (FGFR1, FGFR2, FGFR3, and FGFR4). The FGF/FGFR pathway plays an important role in cancer development and progression by modulating a variety of biological processes, including cell proliferation, survival, and migration [28, 29]. FGFR substrate 2 (FRS2/FRS2α), also known as FGFR-signaling adaptor SNT1 (suc1-associated neurotrophic factor target 1), is regarded as the ‘conning center’ for intracellular signaling elicited by the activation of FGFRs at the cell surface. FRS2 forms complexes with Grb2-Sos and Grb2-Gab1 to activate the Ras/Raf/MEK/ERK and PI3K/Akt pathways [29, 30]. Although FRS2 expression is not regulated by androgen [31], androgen-sensitive prostate cancer cells express FGF2, and its expression is upregulated in response to androgen stimulation [32]. Thus, androgen regulates the activity of FGFR signaling in prostate cancer cells.

In this study, we report for the first time that PKD1 was tightly regulated by androgen at the transcriptional level in prostate cancer cells and was a novel androgen-repressed gene. Inhibition or knockdown of androgen receptor (AR) blocked androgen depletion-induced PKD1 expression, indicating that AR was required for the repression of *PRKD1* gene expression. Further analysis identified FRS2 as a novel mediator of androgen-induced PKD1 repression. The regulation of PKD1 by androgen and AR may have important implications in the therapeutic response to AR-targeted agents.

## RESULTS

### Androgen repressed PKD1 expression in androgen-sensitive prostate cancer cells

**Figure 1:**
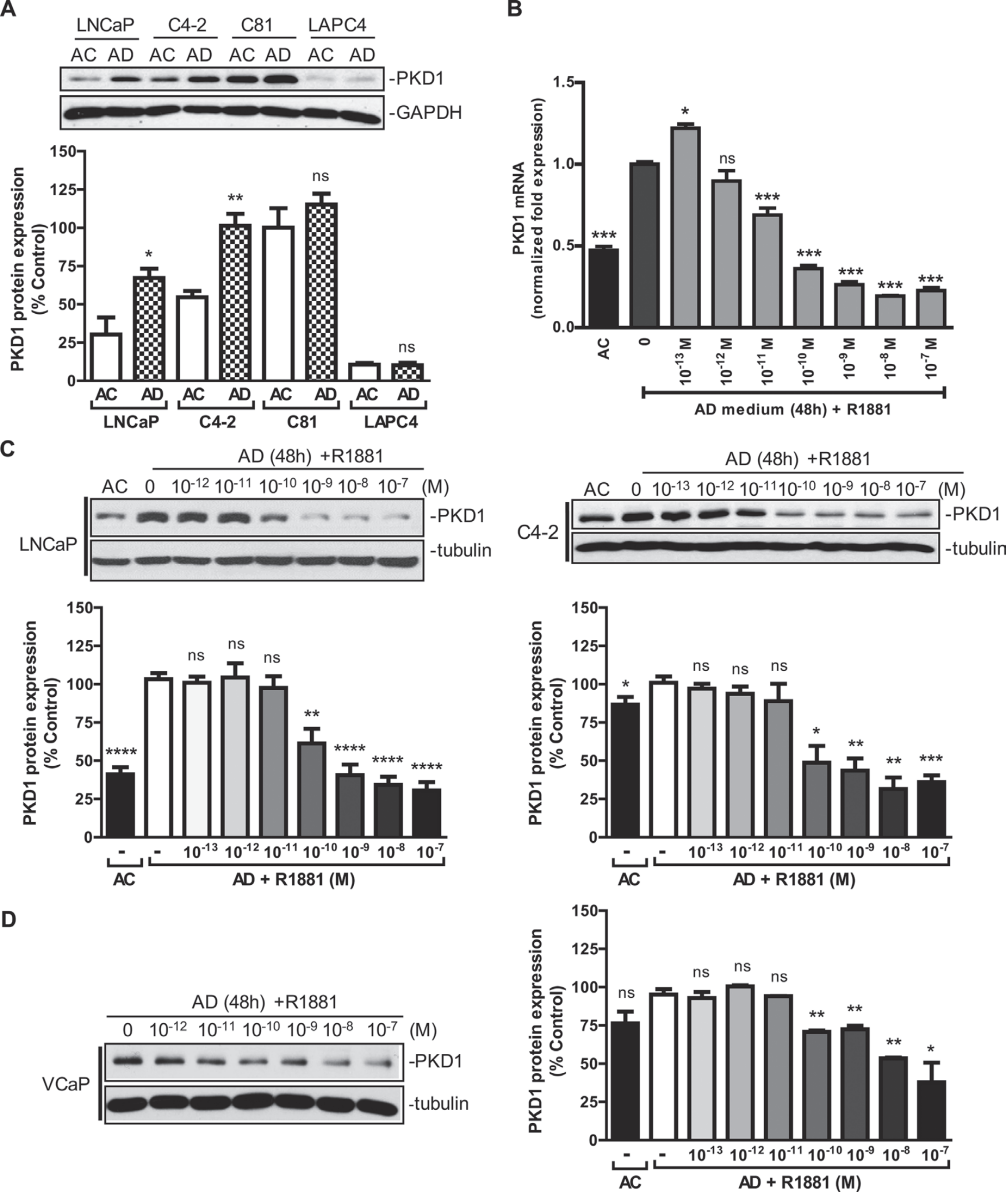
Androgen repressed PKD1 expression (**A**) Effects of androgen depletion on PKD1 expression in prostate cancer cells. LNCaP, C4-2, C81, and LAPC4 cells were grown for 48 h in normal androgen-containing (AC) or androgen-depleted (AD) medium supplemented with charcoal-stripped FBS. Cells were lysed and subjected to immunoblotting for PKD1 and GAPDH (loading control). *Bottom*, quantitative measurement of band intensity by densitometry analysis. The data were expressed as % control with C81 (AC) set as 100%. Data are the mean ± SEM of four independent experiments. (**B**) Androgen inhibited PKD1 transcription. Total RNAs from LNCaP were extracted, and real-time RT-qPCR was conducted using specific PKD1 primers. *GAPDH* was used as internal control. Data are the mean ± SEM of three independent experiments. (**C**) Androgen suppressed PKD1 protein expression. LNCaP and C4-2 cells were grown in androgen-depleted medium for 48 h, following by treatment without or with increasing concentrations of androgen R1881. Cells were harvested after 24 h and subjected to immunoblotting. *Bottom*, the band intensity was quantified by densitometry analysis, and data are the mean ± SEM of ten (LNCaP) or three (C4-2) independent experiments. (**D**) Androgen suppressed PKD1 protein expression in castration-resistant VCaP cells. VCaP cells were grown in androgen-depleted medium for 48 h, followed by treatment without or with androgen R1881 for 24 h. Cells were harvested for immunoblotting. Data from one of three independent experiments are shown. *Right*, quantitative measurement of band intensity from three experiments is shown. ns, not significant; *p < 0.05; **p < 0.01; ***p < 0.001; ****p < 0.0001.

Androgen signaling plays a crucial role in prostate cancer initiation and progression. In this study, we sought to determine whether androgen modulated PKD1 expression and signaling. PKD1 was detected in androgen-sensitive LNCaP cells and two castration-resistant LNCaP-derivative cell lines, C4-2 (androgen-hypersensitive) and C81 (androgen-insensitive), but not in androgen-sensitive LAPC4 cells. As shown in Figure 1A, a significant increase in PKD1 expression was observed upon androgen depletion (AD) in LNCaP and C4-2 cells and to a lesser extent in C81 cells. R1881, a synthetic androgen agonist, induced remarkable concentration-dependent suppression of PKD1 expression at the transcript (Figure 1B) and protein (Figure 1C) levels in LNCaP and C4-2 cells. R1881 also suppressed PKD1 expression in VCaP cells, a castration-resistant prostate cancer cell line that expresses wild-type AR, in a concentration-dependent manner (Figure 1D). Interestingly, PKD2 expression was similarly suppressed by R1881 in a concentration-dependent manner in LNCaP and VCaP cells (Supplementary Figure 1A–1B). PKD3 was also upregulated upon androgen withdraw in LNCaP cells, despite its low endogenous expression (Supplementary Figure 1A). In contrast, androgen did not affect the expression of PKD1 and PKD2 in another castration-resistant cell line, 22Rv1, which expresses both full-length AR and truncated AR variants (Supplementary Figure 1C), suggesting that the effect of androgen may be cell context-dependent. Taken together, we concluded that PKD1 was an androgen-repressed gene.

### PKD1 expression was dependent on the induction of a repressor protein

**Figure 2:**
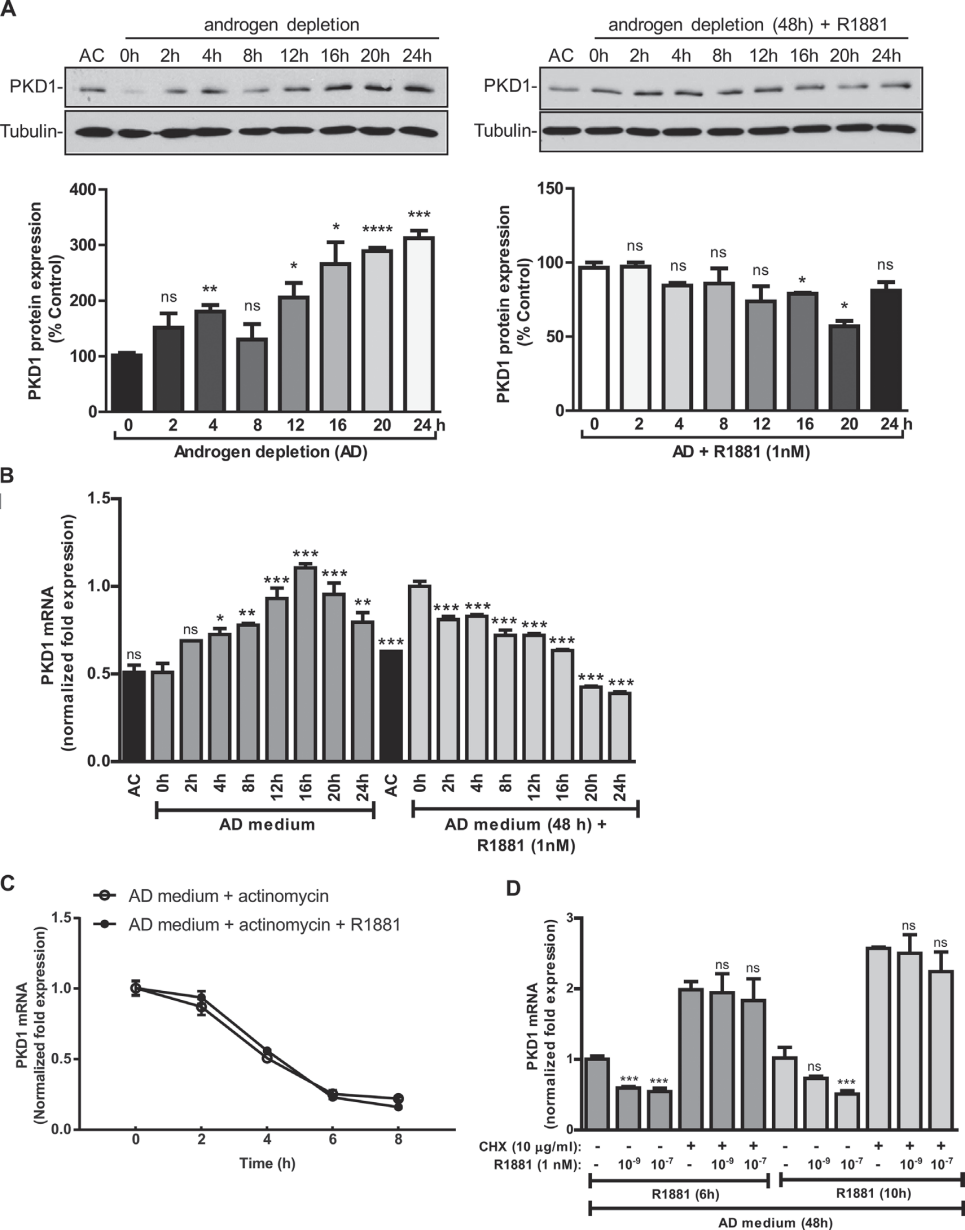
PKD1 expression was dependent of the induction of a repressor protein (**A**) Kinetics of PKD1 regulation by androgen. *Left panels*, LNCaP cells were grown in AD medium for the indicated times. *Right panels*, LNCaP cells were grown in AD medium for 48 h, followed by treatment with R1881 (1 nM) for the indicated times. Cells were harvested and subjected to immunoblotting for PKD1 and tubulin (loading control). Cells grown in AC medium were used as the control. Representative data from one of four experiments are shown. *Bottom*, quantitative measurement of band intensity by densitometry analysis. Data are the mean ± SEM of three independent experiments. (**B**) Kinetics of PKD1 transcript expression. LNCaP cells were treated as above in “A”. Total RNAs were extracted, and the kinetics of PKD1 mRNA induction/suppression were examined by real time RT-qPCR. (**C**) R1881 did not affect PKD1 mRNA stability. LNCaP cells were grown either in AD medium for 48 h, followed by the addition of actinomycin D (2 ng/mL) with or with R1881 (1 nM) for the indicated times. Total RNAs were extracted and subjected to real time RT-qPCR for analysis of PKD1 transcripts. Not significant by paired t test (*p* > 0.5). (**D**) PKD1 expression required the induction of a repressor protein. LNCaP cells were grown in AD medium for 48 h, followed by R1881 treatment with or without cycloheximide (CHX) for 6 or 10 h. Total RNAs were extracted, and the levels of PKD1 mRNA were measured by real-time RT-qPCR. *GAPDH* was used as a loading control. Data are the mean ± SEM of at least three independent experiments. ns, not significant; *p < 0.05; **p < 0.01; ***p < 0.001; ****p < 0.0001.

The kinetics of PKD1 regulation in response to androgen deprivation or R1881 treatment was examined. As shown in Figure 2A, androgen deprivation gradually up regulated PKD1 protein expression, which peaked at 16–24 h, while R1881 suppressed PKD1 expression with similar kinetics. The induction of PKD1 transcript and its inhibition by R1881 correlated well with the time-course of protein expression (Figure 2B).

To gain insights into the regulation of PKD1 by androgen, we first examined whether R1881 affected PKD1 mRNA stability. The half-life (t½) of PKD1 mRNA was determined in the presence of actinomycin D, an inhibitor of gene transcription. As shown in Figure 2C, the t½ of PKD1 mRNA was about 4 h, which was not significantly altered by the addition of R1881 (*p* > 0.5), indicating that R1881 did not impact the stability of PKD1 mRNA. Next, cycloheximide (CHX) was used to inhibit protein synthesis to determine whether the regulation of PKD1 gene expression by androgen involved *de novo* protein synthesis. CHX induced a nearly 2-fold increase in PKD1 expression and completely blocked R1881-induced PKD1 downregulation, indicating that the suppression of PKD1 expression likely required the induction of a repressor protein (Figure 2D). This finding was in line with the gradual onset of PKD1 regulation by androgen, further supporting the involvement of a repressor protein. Taken together, our data indicated that androgen-regulated PKD1 expression was dependent on the presence of a repressor protein.

### AR mediated PKD1 repression by androgen

**Figure 3:**
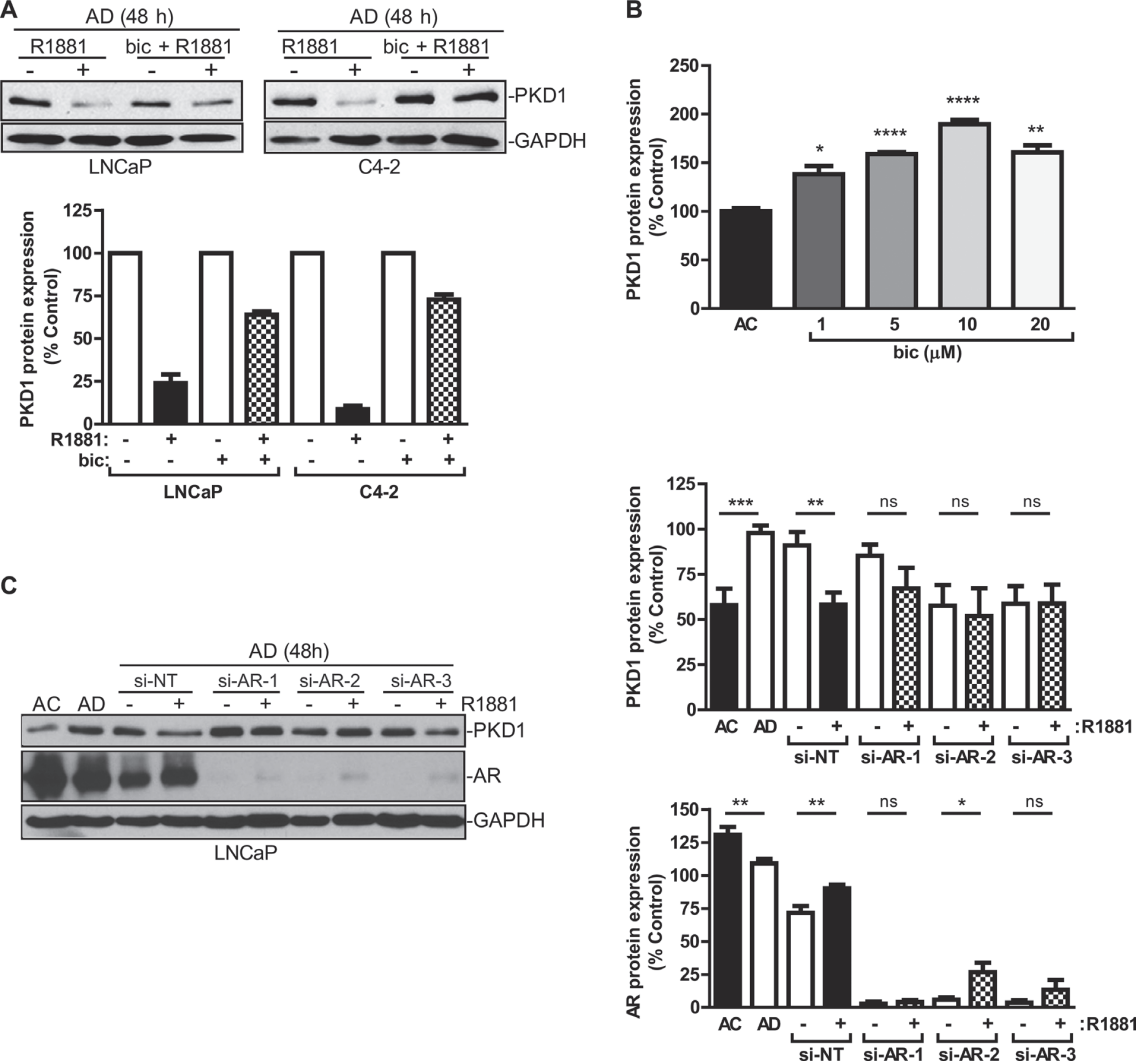
AR mediated PKD1 repression by androgen (**A**) AR inhibition led to increased PKD1 expression. LNCaP and C4-2 cells cultured in AD medium for 48 h were treated with or without R1881 (1 nM) ± bicalutamide (Bic) (10 μM) for 16 h. *Bottom*, quantitative measurement of band intensity from two experiments. (**B**) Bicalutamide caused PKD1 upregulation. LNCaP cells were treated with increasing concentrations of bicalutamide for 48 h, followed by immunoblotting for PKD1. The band intensity was quantified by densitometry analysis, and data are the mean ± SEM of three independent experiments. (**C**) AR was required for transcriptional regulation of PKD1 by androgen. LNCaP cells were transfected with non targeting siRNA (si-NT) or AR siRNAs (si-AR-1, -2, -3). After 48 h, the medium was replenished with AD medium with or without R1881 (1 nM) for 16 h. Cells were collected and subjected to immunoblotting for PKD1, AR, and GAPDH. *Right*, quantitative measurement of band intensity for PKD1 (*top*) and AR (*bottom*) from three experiments is shown. ns, not significant; *p < 0.05; **p < 0.01; ***p < 0.001; ****p < 0.0001. Data are the mean ± SEM of six independent experiments.

Androgens are important hormones for normal physiology and are responsible for certain disease conditions. Their actions are mediated by the AR, a ligand-dependent nuclear transcription factor. Androgens binds to AR after entering the cells to form an androgen-receptor complex, which then translocates to the nucleus where it binds to androgen response elements (AREs) in the promoter regions and regulates the transcription of its target genes. The actions of AR can be blocked by AR inhibitors, such as bicalutamide (Casodex) or enzalutamide (MDV3100). Bicalutamide is known to bind AR and leads to the formation of a transcriptionally inactive AR complex [33]. In this study, we sought to examine whether AR was required for the repression of PKD1 expression by R1881, and bicalutamide was used to determine whether the inhibition of AR activity affected PKD1 expression. After androgen deprivation, LNCaP and C4-2 cells were treated with R1881 at 1 nM in the presence or absence of bicalutamide (10 μM). As shown in Figure 3A, bicalutamide significantly reversed R1881-induced PKD1 repression in LNCaP and C4-2 cells. In LNCaP cells, inhibition of AR by bicalutamide also upregulated PKD1 protein expression in a concentration-dependent manner (Figure 3B). The specific role of AR was then examined using multiple AR-targeted siRNAs. Our data showed that knockdown of AR by three siRNAs targeting different regions of the AR transcript significantly blocked R1881-induced PKD1 suppression in LNCaP (Figure 3C) and C4-2 cells (data not shown). AR knockdown was confirmed by western blotting. Taken together, these data suggested that AR was required for the transcriptional repression of PKD1 gene expression caused by androgen stimulation.

To determine whether AR directly regulated the expression of PKD1, we analyzed the promoter region of PKD1, which led to the identification of two potential AREs upstream of the transcription start site (TSS). The human PKD1 gene spans ~45.7 kb. Analysis of up to 5000 bp of the promoter region upstream from the TSS revealed two putative AREs. (ARE1, 5′-AGTACTTTAAGCTCT-3′; ARE2, 5′-AGAACAAAATAAGCT-3′; (Supplementary Figure 2A). The regions (pm1 and pm2) that contained the AREs were separately cloned into the pTA-Luc reporter. Their activities were analyzed in LNCaP cells cultured in the presence or absence of androgen depletion, followed by treatment with or without R1881. Our data indicated that no luciferase activity was detected from both reporters in LNCaP cells (Supplementary Figure 2B), implying that the AREs in PKD1 promoter did not play an active role in regulating PKD1 transcription in response to androgen stimulation.

### An AR co repressor screen revealed FRS2 as the potential mediator of androgen-induced PKD1 repression

**Figure 4:**
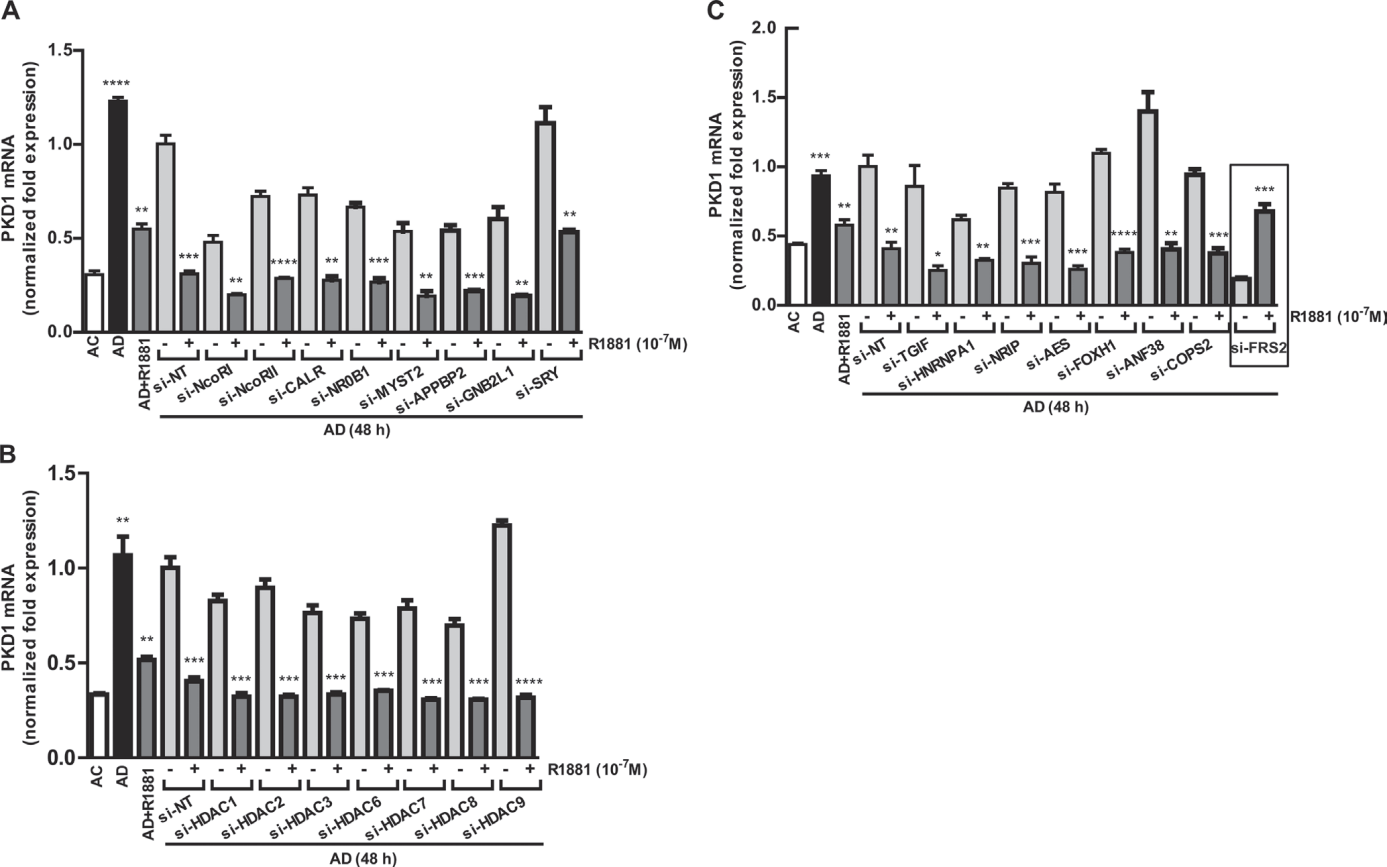
Screening of AR corepressors (**A**–**C**) An esiRNA screen that targeted 23 AR corepressors and other related proteins was conducted in LNCaP cells. The cells transfected with esiRNAs were subjected to androgen depletion for 48 h, followed by treatment with or without R1881. Levels of PKD1 transcript was analyzed by real time RT-qPCR. Non targeting siRNA (si-NT) was used as the control. Student's *t*-tests were used to determine the statistical significance between the untreated and R1881-treated groups within each pair of esRNA knockdown samples. *p < 0.05; **p < 0.01; ***p < 0.001.

**Figure 5:**
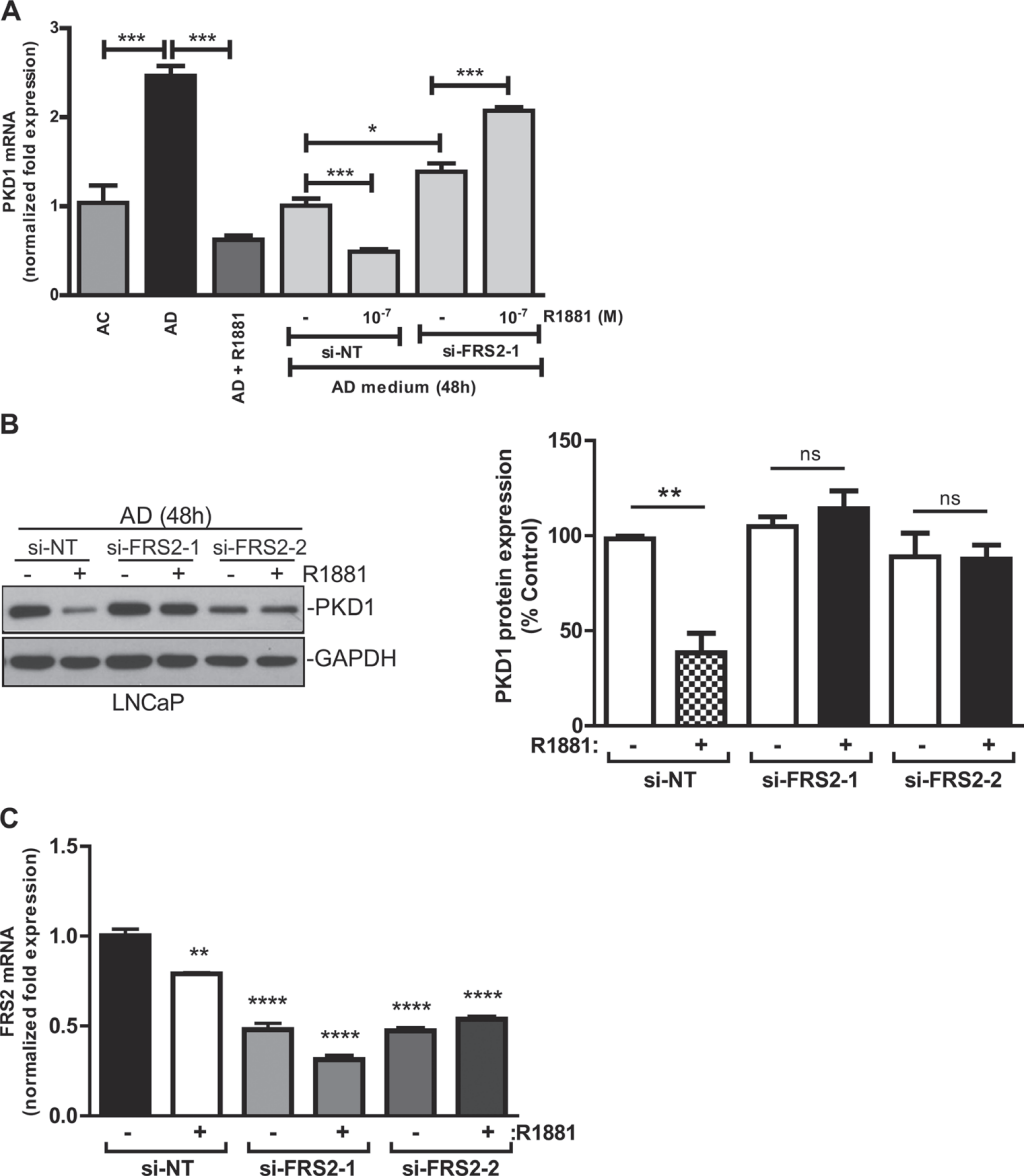

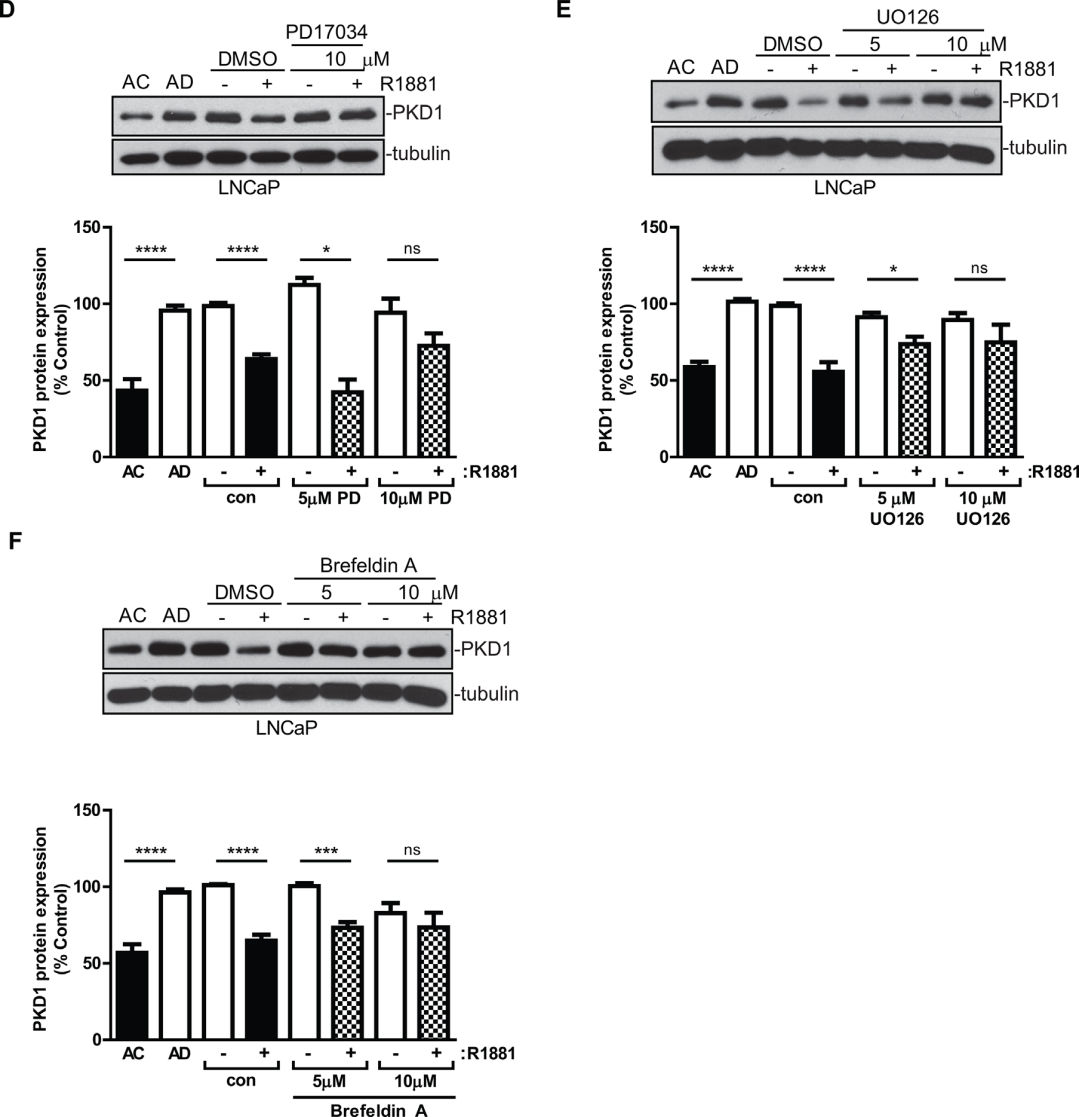
FRS2 was required for androgen-induced PKD1 repression (**A**) Knockdown of FRS2 reversed androgen-induced repression of PKD1 transcription. Cells were transfected with FRS2 siRNA (si-FRS2-1) and a non targeting siRNA (si-NT), followed by treatment with or without R1881. PKD1 transcripts were analyzed by real-time RT-qPCR. Representative data from one of three independent experiments with triplicate measurements are shown. (**B**) Knockdown of FRS2 blocked the repression of PKD1 protein by androgen. LNCaP cells were transfected with two different FRS2 siRNAs (si-FRS2-1, -2), followed by treatment with R1881. *Right*, quantitative measurement of band intensity for PKD1 from three experiments is shown. (**C**) Real-time RT-PCR confirmed the knockdown of FRS2. Cells from “B” were subjected to RNA extraction, followed by real-time RT-PCR for levels of PKD1 transcript. (**D**–**F**) Androgen repression of PKD1 was dependent on a secretory pathway involving FGFR and MEK. LNCaP cells were grown in AC or AD medium for 48 h, followed by treatment with or without R1881 in the presence or absence of the FGFR inhibitor PD17034 (D), the MEK inhibitor UO0126 (E), and brefeldin A (F) for 16 h. Cell lysates were subjected to immunoblotting for PKD1. Representative images from one of at least three independent experiments are shown. *Bottom*, quantitative measurement of band intensity by densitometry analysis. Data are the mean ± SEM of five to seven independent experiments.

The involvement of AR and an androgen-induced repressor protein prompted us to conduct an esiRNA screen that targeted 23 AR corepressors and other related proteins. LNCaP cells were transfected individually with 23 esiRNAs, followed by androgen depletion and treatment with or without R1881. Levels of PKD1 transcript were analyzed by real time RT-qPCR. In the controls, androgen depletion induced PKD1 expression, and treatment with R1881 caused over 2-fold reduction in PKD1 mRNA. As shown in Figure 4, similar to the non targeting siRNA, R1881-induced PKD1 repression was not affected by the depletion of all target genes, with the exception of FRS2. Knockdown of FRS2 by esiRNA completely reversed the repression of PKD1 transcription by R1881 (Figures 4C, 5A). In summary, FRS2 was identified as a potential repressor of PKD1 gene expression.

### Androgen repressed PKD1 expression through a FGFR/FRS2/MEK/ERK pathway

The role of FRS2 was further validated using FRS2 siRNAs (si-FRS2-1, -2). Depletion of FRS2 abolished the R1881-induced suppression of PKD1 transcription, confirming FRS2 as a potential mediator of androgen-dependent PKD1 repression (Figure 5A). Furthermore, at the protein level, knockdown of FRS2 by two different siRNAs completely abrogated the downregulation of PKD1 by R1881 (Figure 5B). *FRS2* siRNAs caused significant knockdown of *FRS2* mRNA (Figure 5C). Thus, FRS2 mediated androgen-induced PKD1 repression.

The adaptor protein FRS2 is a major mediator of the FGFR signaling in normal and malignant cells. FGFR stimulation by FGF leads to the tyrosine phosphorylation of FRS2, which then forms a complex with Grb2 and Sos to activate the downstream Ras/Raf/MEK/ERK signaling pathway. Androgen-sensitive LNCaP cells express low levels of FGF2, and its expression is upregulated in response to androgen stimulation [32]. Here, we sought to determine whether the FRS2-mediated FGFR signaling pathway was involved in the regulation of PKD1 by androgen. As shown in Figure 5D, PD173074, an inhibitor of FGFR, significantly reversed R1881-induced PKD1 repression, indicating that FGFR activity was required for the inhibition of PKD1 by androgen/AR. Next, the role of the FGF-activated MEK/ERK MAPK signaling pathway was evaluated. Our data demonstrated that R1881-induced PKD1 suppression was abrogated in a concentration-dependent manner by UO126, a MEK inhibitor. Thus, MEK/ERK activity was also required for the suppression of PKD1 by androgen (Figure 5E). Since the suppression of PKD1 is likely associated with the secretion of a FGFR ligand, we tested the effects of inhibiting secretory pathways on the expression of PKD1 using brefeldin A (BFA), a fungal metabolite and an inhibitor of intracellular protein transport that inhibits constitutive secretion from the *trans*-Golgi network. Our data indicated that BFA at 5 and 10 μM completely reversed androgen-induced PKD1 suppression (Figure 5F). In summary, our data implied that androgen suppressed PKD1 expression through an indirect FGFR/FRS2/MEK/ERK pathway in prostate cancer cells.

## DISCUSSION

In this study, we present the novel findings demonstrating that PKD1 was repressed by androgen/AR at the mRNA and protein levels in androgen-sensitive prostate cancer cells, identifying PKD1 as a novel androgen-repressed gene. We further identified FRS2 as a novel mediator of androgen-induced repression of PKD1 expression. The cross-regulation of PKD1 by androgen/AR places PKD1 in the AR-induced signaling network, which is critical to prostate cancer progression. The transcriptional regulation of PKD isoforms has not been studied in the past. Our study provides the first mechanistic understanding of a novel androgen-induced AR/FRS2/MEK/ERK pathway that regulates the expression of PKD1. As a well-documented prosurvival signaling protein, PKD1 upregulation in response to androgen deprivation and anti-androgen treatment may have significant implications in therapy resistance and progression to CRPC.

The class I steroidal nuclear receptor AR is a critical regulator of tumor initiation and progression in both early and advanced prostate cancer. As a transcription factor, AR exerts its actions mainly through regulating the expression of a host of target genes. Among them, AR-stimulated genes have been extensively studied, with prostate-specific antigen (PSA) being the best characterized. In contrast, AR-repressed target genes have not been well characterized. These genes constitute a large portion of AR-targeted genes, and some have been shown to play essential roles in prostate cancer progression [34, 35]. Diverse mechanisms have been proposed to account for the repression of target gene expression by AR. These include both genomic mechanisms, such as active repression via the recruitment of corepressor complexes, and nongenomic mechanisms, such as regulation of signaling pathways [34]. Our data showed that inhibition or silencing of AR blocked the suppression of PKD1 by R1881, indicating that AR was required for the downregulation of PKD1. Initially, analysis of the 5′ promoter region of the PKD1 gene led to the identification of two potential AREs, which prompted us to investigate the direct role of AR in transcriptional repression of the PKD1 gene. However, analysis of the transcriptional activity of the ARE-containing PKD1 promoter failed to detect any androgen-induced transcriptional activities associated with pm1 and pm2, suggesting that the identified potential AREs may be inactive. Although less common, inactive AREs have been demonstrated, even in the presence of AR binding, and more complex mechanisms have been suggested to be involved in the regulation of genes nearby these AREs in prostate cancer cells [36, 37]. Importantly, kinetic analysis demonstrated a slow and gradual onset of PKD1 downregulation at the protein and transcript levels, which peaked at about 16–20 h in response to androgen; this finding also provides evidence against a mechanism involving active transcriptional repression through direct interaction with AREs. The involvement of an androgen-induced repressor is supported by the data showing that CHX abolished androgen-induced PKD1 repression. Although this may also occur without the synthesis of a repressor, for example, the suppressive effects of AR could be mediated through its interaction with a pre-existing labile protein at the AR-repressed loci; CHX treatment will similarly abolish the repressive effect mediated by this labile protein. Certainly, our findings do not exclude the possibility that there may be distal ARE sites that bind to AR and contribute to AR-mediated PKD1 repression. Overall, our current data support an AR-mediated indirect mechanism involving the cell surface adaptor protein FRS2 in the repression of PKD1 by androgen. These findings were based on an unbiased RNAi screen of a library of AR corepressor proteins. Further analysis validated the role of FRS2, as well as its upstream FGFR and the downstream MEK/ERK pathway, in the regulation of PKD1 by androgen.

In androgen-sensitive prostate cancer cells, depletion of FRS2 blocked R1881-induced PKD1 suppression at both the transcriptional and protein levels. Additionally, inhibition of FGFR and MEK, as well as protein secretion, blocked R1881-induced repression of PKD1. Thus, androgen may repress PKD1 through an AR-induced FGFR/FRS2/MEK/ERK pathway to inhibit PKD1 expression in prostate cancer cells. A previous study showed that FRS2 expression is not regulated by androgen in LNCaP cells [31]. However, in androgen-sensitive LNCaP cells, low levels of FGF2 are detected, and the expression of FGF2 is upregulated in response to androgen stimulation [32]. Additionally, androgen stimulates the activity and production of FGF2 and FGF-binding protein in PC3 prostate cancer cells with stably overexpressed AR [38]. In a different study, however, Kassen et al. showed that FGF2 is not expressed, and androgen in turn acts by increasing the bioavailability of FGF2 by releasing trapped FGF2 from the extracellular matrix through activation of heparinase, which leads to activation of FGFR and stimulation of LNCaP cell proliferation [39]. Regardless of these discrepancies, in all cases, AR promotes the activation of FGFR in prostate cancer cells, which results in phosphorylation of FRS2 and activation of the downstream Ras/Raf/MEK/ERK signaling pathway. By inhibiting MEK activity, we confirmed the requirement for MEK/ERK signaling activity in the suppression of PKD1 by R1881. This evidence supports the notion that PKD1 is repressed by an AR-induced FGFR/FRS2/MEK/ERK pathway in androgen-sensitive prostate cancer cells. The binding of FGF to FGFR leads to the recruitment of multiple adaptor proteins, including FRS2, Grbs, Sos, and Gab1, and induces the activation of multiple downstream signaling pathways, including MEK/ERK, PI3K/Akt, PLCγ/PKC, and Stat3 pathways. We must state that although our data demonstrated a major role of the MEK/ERK pathway in the regulation of PKD1 expression by androgen, our data did not completely exclude the potential involvement of other pathways, which will be investigated in our future studies.

Our study identified PKD1 as an androgen/AR-repressed gene and uncovered a novel indirect mechanism through which AR regulates PKD1 expression. Although the functional implication of this regulation in prostate cancer progression is still unclear, PKD1 is an important prosurvival signaling protein in normal and cancer cells that functions by regulating multiple signaling pathways, such as stimulating NF-κB, ERK1/2, and Akt and inhibiting JNK and p38 [20, 25, 40]. This notion is further supported by our previous findings that PKD1 protects androgen-sensitive LNCaP prostate cancer cells from phorbol ester-induced apoptosis [25]. Thus, the upregulation of PKD1 as a result of inhibition or loss of AR may promote tumor cell survival and contribute to therapeutic resistance to AR-targeted agents. This further implies that PKD may represent a viable target for mitigating therapy resistance. In castration-resistant C81, 22Rv1, and VCaP cells, we observed different responses to androgen in terms of PKD1 regulation; although androgen did not affect PKD1 expression in 22Rv1 cells, VCaP cells, which express wild-type AR, did respond to androgen stimulation by downregulating PKD1 in a concentration-dependent manner, and minor effects were also observed in C81. This cell context-dependent responsiveness to androgen may be linked to the activity of the AR/FGFR/FRS2 signaling pathway and variations in the expression of its signaling components.

In summary, our study identified PKD1 as a novel androgen/AR-suppressed gene. The suppression of PKD1 was mediated through an indirect mechanism that involved FRS2, a cell surface adaptor protein that connects FGF/FGFR to the downstream MEK/ERK signaling pathway. Our findings suggested that the prosurvival function of PKD1 may have significant implications in prostate cancer progression and therapy resistance. PKD1 may be targeted to enhance the therapeutic response to anti-androgens in prostate cancer treatment.

## MATERIALS AND METHODS

### Reagents and antibodies

The synthetic androgen methyl trienolone (R1881) was obtained from Perkin-Elmer Life Sciences (Boston, MA), and bicalutamide was purchased from Enzo Life Sciences (Farmingdale, NY). Charcoal-treated fetal bovine serum (FBS) was from Hyclone (Logan, UT) and Sigma (St. Louis, MO). Other cell culture reagents and media were from American Type Culture Collection (ATCC; Rockville, MD). Anti-PKD1, anti-PKD2, and anti-AR antibodies were purchased from Cell Signaling Technology (Danvers, MA). Antibodies targeting GAPDH and α-tubulin were from Santa Cruz Biotechnology, Inc. (Santa Cruz, CA). Goat anti-rabbit and goat anti-mouse horseradish peroxidase-conjugated secondary antibodies were from Promega (Madison, WI).

### Cell culture and siRNA transfection

LNCaP, C4-2, C81, VCaP, and PC-3 cells were obtained from ATCC (Manassas, VA) and were cultured according to the manufacturer's recommendations. LNCaP cells were discarded after 12 passages. The 23 AR corepressor esiRNAs were obtained from Sigma-Aldrich (Supplementary Table 1). The non targeting siRNA and AR and FRS2 siRNAs were obtained from Integrated DNA Technologies (Coralville, IA). The esiRNAs and siRNAs were transfected into cells using DharmaFECT reagent according to the manufacturer's instructions (GE Dharmacon, Lafayette, CO).

### Western blot analysis

Cells were collected and lysed in IP lysis buffer (50 mM Tris-HCl, pH 7.4, 150 mM NaCl, 1.5 mM MgCl_2_, 10% glycerol, 1% Triton X-100, 5 mM EGTA, 20 μM leupeptin, 1 mM AEBSF, 1 mM NaVO_3_, 10 mM NaF, and 1× protease inhibitor cocktail). Lysate protein concentrations were determined using the Pierce BCA Protein Assay Kit (Thermo Fisher, Hudson, NH). Cell lysates were separated by SDS-PAGE and transferred to nitrocellulose membranes. After blocking with 5% milk, the membranes were incubated with primary antibodies in blocking buffer at 4°C overnight. After washing, the membranes were incubated with secondary antibodies at room temperature for 1 h. Protein bands were detected using an enhanced chemiluminescence (ECL) kit. Anti-α-tubulin or anti-GAPDH antibodies were used as a loading control. Densitometry analyses were performed with ImageJ software (NIH).

### Real-time RT-PCR

Total RNAs were isolated from LNCaP cells using a RNeasy kit (Qiagen, Valencia, CA) according to the manufacturer's protocol. One microgram of total RNAs was used to generate cDNA using an iScript cDNA synthesis kit. Real-time PCR was subsequently performed using SsoFast EvaGreen Supermix on a CFX96 Real-Time PCR Detection System (Bio-Rad, Richmond, CA, USA). The following primers were used: PKD1, forward primer 5′-CGCACATCATCTG CTGAACT-3′ and reverse primer 5′-CTTTCGGTGCA CAACGTTTA-3′; FRS2, forward primer 5′-ATGG GAATGAGTTAGGTTCTGGC-3′ and reverse primer 5′-GCGGGTGTATAAAATCAGTTCTGTG-3′. Data were normalized automatically by using GAPDH as the loading control, with the following primers: forward primer 5′-GCAAATTCCATGGCACCGT-3′ and reverse primer 5′-TCGCCCCACTTGATTTTGG-3′.

### Statistical analysis

All statistical analyse were carried out using GraphPad Prism IV software. A *p* value of less than 0.05 was considered statistically significant (*p < 0.05; **p < 0.01; ***p < 0.001; ****p < 0.0001).

## SUPPLEMENTARY MATERIALS FIGURES AND TABLE




