# Supplementary material for: Androgen suppresses protein kinase D1 expression through fibroblast growth factor receptor substrate 2 in prostate cancer cells
Source: Oncotarget. 2017 Jan 6;8(8):12800–11. doi: 10.18632/oncotarget.14536 (PMC5355056; doi:10.18632/oncotarget.14536)
Supplement: Supplementary file 1 [file oncotarget-08-12800-s001.pdf]

# Androgen suppresses protein kinase D1 expression through fibroblast growth factor receptor substrate 2 in prostate cancer cells

## Supplementary Materials

### Supplementary Information

### Generation of the PKD1 reporter gene constructs

Two potential AR binding sites (ARE1 and ARE2) were identified through a promoter analysis. As shown in Supplementary Figure S2A, the pm1 promoter region located between –3253 to –2485 (769 bp) contains ARE1 with the sequence "–3090 agtactttaagctct –3076" upsteam of TSS, while pm2 located between –2484 to –1380 (1106bp) contains ARE2 with the sequence "–1478 agaacaaaataagct –1464" upsteam of TSS. Both regions were PCR-amplified separately and subcloned into the pTA-Luc vector at the KpnI/NcoI sites, resulting in the pTA-Luc-pm1 and pTA-Luc-pm2 reporter constructs. Note that the region immediately upstream of the TSS (–1400 to 0) was not analyzed since it did not contain any predicted AREs.

### Luciferase reporter gene assay

One day before transfection, LNCaP cells were plated at a density of $3 \times 10^5$/well on a 12-well plate. Cells were transfected separately with 1.5 µg/well of the pTA-Luc-pm1 and pTA-Luc-pm2 firefly luciferase reporter, along with 300 ng/well of pTK-RL renilla luciferase plasmid for normalization using Lipofectamine 2000 (Invitrogen, Carlsbad, CA). Twenty-four hours after transfection, cells were washed three times and cultured in androgen-depleted (AD) or -containing (AC) medium for 48 h, followed by treatment with or without R1881 for another 16 hours. The cells were then collected, lysed, firefly luciferase activity was analyzed and normalized to renilla luciferase activity using the dual-luciferase reporter assay system (Promega, Madison, WI) according to manufacturer's instructions. Cells transfected with the pTA-Luc-NF-κB reporter and treated with or without PMA at 10 nM for 6 h were assayed as the positive control.

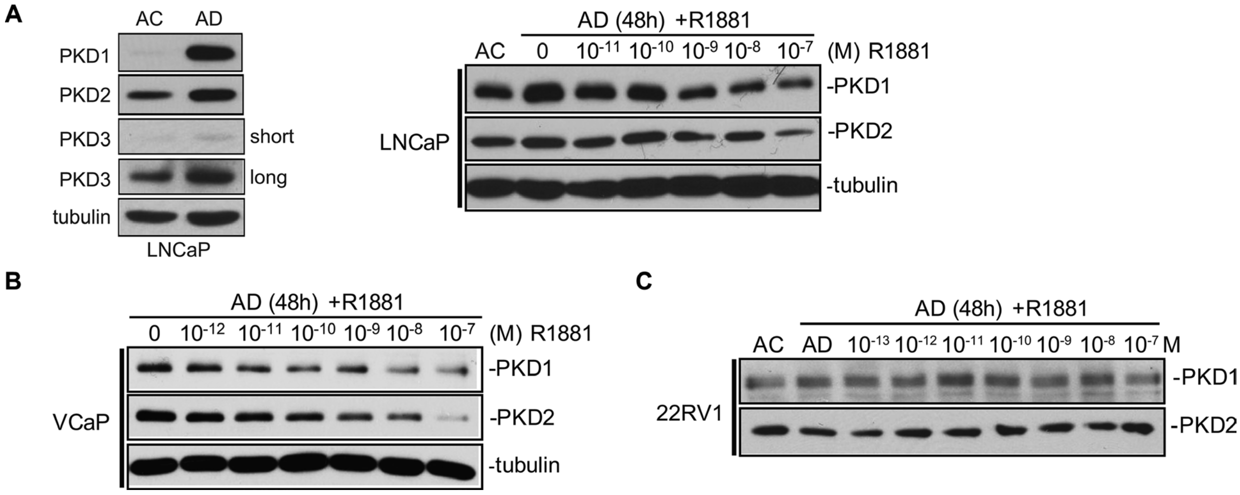


**Supplementary Figure 1: Effects of androgen on PKD protein expression in prostate cancer cells.** LNCaP cells were grown in AC and AD medium for 4 days. The cells were harvested and subjected to immunoblotting for PKD isoforms (**A**, *left*). *short* or *long* indicates western blot exposure times. LNCaP (A, *right*), VCaP (**B**) and 22Rv1 (**C**) prostate cancer cells were grown in AD medium for 48 h, followed by treatment without or with increasing concentrations of R1881 for 24 h. Cells were harvested for immunoblotting of PKD1 and PKD2 with α-tubulin blotted as loading control. The above experiments were repeated at least three times and data from a representative experiment are shown.

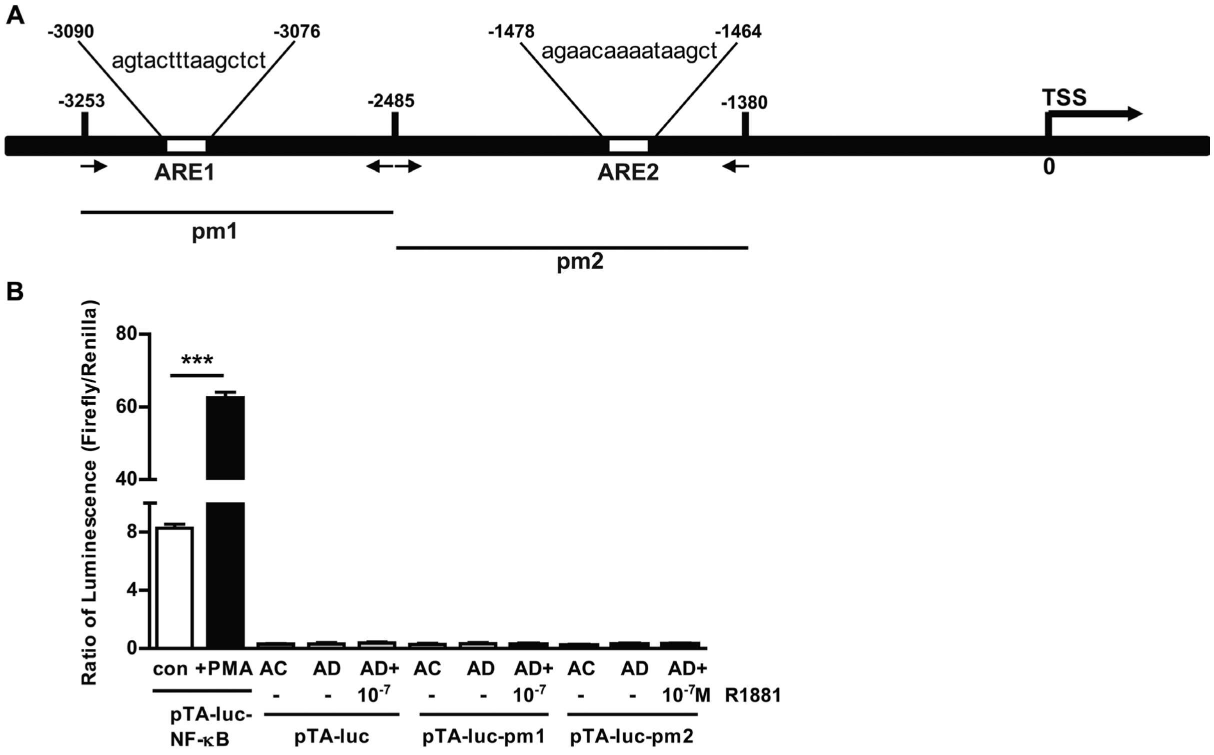


**Supplementary Figure 2: AREs in the promoter region of PKD1 were not sensitive to androgen.** (**A**) A schematic map of the PKD1 5'-promoter region. Sequences and locations of putative AREs, ARE1 and ARE2 are indicated. (**B**) The two AREs were not active transcriptionally. Regions of the PKD1 promoter that contains ARE1 (pm1) and ARE2 (pm2) were separately cloned into pTA-luc vector to give rise to pTA-Luc-pm1 and pTA-Luc-pm2. The two reporter plasmids were separately transfected into LNCaP cells along with an internal control plasmid pTK-RL. Twenty-four hours after transfection, the medium was changed to androgen-depleted (AD) or -containing (AC) medium for 48 h, followed by treatment with or without R1881 for another 16 hours. As controls, the cells were transfected with pTA-Luc-NF-κB cells and subjected to PMA treatment at 10 nM for 6 h. The cells were then harvested and luciferase activity was measured. Results are the mean ± SEM from three independent experiments. ***$p < 0.001$.

**Supplementary Table 1: List of the AR corepressor esiRNAs and their cDNA target sequences.**
See Supplementary_Table_1
